# OA10 Sequential biologic usage in refractory psoriatic arthritis using ultrasound to detect psoriatic arthritis specific sub-clinical active disease

**DOI:** 10.1093/rap/rkac066.010

**Published:** 2022-09-28

**Authors:** Afzal Latheef, Hashmi Hashim, Rizwan Rajak

**Affiliations:** St George's University Hospitals NHS Foundation Trust, London, United Kingdom; King's College Hospital NHS Foundation Trust, London, United Kingdom; Croydon Health Services NHS Trust, London, United Kingdom

## Abstract

**Introduction/Background:**

Psoriatic arthritis is a heterogenic, immune-mediated musculoskeletal disease that affects joints, enthesis, and axial skeleton, and is associated with cutaneous psoriasis.

With the advancement in therapeutics, biological agents have shown marked improvement in multiple domains of the disease. Many studies have proven sequential biologic switching has better persistence and overall improvement in quality of life. Musculoskeletal Ultrasound has the potential to become a rheumatologist’s stethoscope, as it can improve clinical evaluation in detecting inflammation and structural damage, and thus treatment response, in psoriatic arthritis.

We present a case where we used both approaches to treat a refractory psoriatic arthritis.

**Description/Method:**

This 39-year-old South African origin gentleman was initially referred to the Rheumatology department in 2013 with throbbing pain in his hands and stiffness in heels associated with scalp and nail changes secondary to psoriasis. His past medical history included psoriasis and sports trauma related left anterior cruciate ligament (ACL) tear. He also had a strong family of psoriasis.

His initial autoimmune results were negative. The Magnetic Resonance Imaging (MRI) of his right ankle at the time, revealed enthesopathy with surrounding oedema, consistent with psoriatic arthropathy.

The patient was managed initially with methotrexate. Over the following months, he experienced significant fatigue and hypersomnia, and discontinued the medication. He re-presented one and half years later with a flare on his hands and feet. The clinical examination showed onycholysis, guttate pattern psoriasis and synovitis in his Proximal Interphalangeal Joints (PIPJ). He was started on Leflunomide, and his symptoms improved, however he suffered loose stools. The drug frequency was tapered, but the efficacy was poor leading to recurrent flares.

Subsequently, he was switched to Adalimumab in 2016 and his symptoms improved significantly. However, he was then switched to Ustekinumab following development of Autoimmune Hepatitis. He was also initiated on sulfasalazine at the same time but was discontinued due to side effects.

In 2018, he started to notice finger swellings especially on the left hand. Clinically there was no swelling and mild tenderness noted. An ultrasound of his hands showed paratenonitis of the left third and fourth proximal interphalangeal joints and the left 1st distal interphalangeal joint consistent with active psoriatic arthritis. He was then switched to Secukinumab which he has remained on with sustained clinical and imaging remission.

**Discussion/Results:**

We present a refractory case of psoriatic arthritis with sequential usage of conventional and biologic DMARDs.

Often, patients fail to respond to first line conventional DMARDs (cDMARDS) and need switching to biologicals due to lack of initial efficiency or loss of efficiency over time or due to adverse effects. A study on sequential biologic usage highlighted drug inefficacy as a major cause (67%), followed by adverse effects (25%). In this case, cDMARDs and Adalimumab were discontinued due to adverse effects, while Ustekinumab was stopped due to secondary failure (inefficacy following an initial period of response). Studies have found an increase in clinical response and better outcomes while switching biologics sequentially, especially to second line therapy making it a valid therapeutic strategy. Studies have shown, Secukinumab is superior as second line therapy to adalimumab, etanercept, infliximab, and ustekinumab.

Also, this case emphasise ultrasound has high reliability and sensitivity in determining early and active disease with features that are particular to Psoriatic Arthritis including tendon thickening, paratenontis and enthesitis, and can differentiate most types of inflammatory arthritis (such as Rheumatoid Arthritis) and  mechanical joint pain. This can also be used as a tool for monitoring drug effectiveness and subclinical improvement. Also, ultrasound enthesitis reflects the disease process in psoriatic arthritis, more accurately than clinical enthesitis. This makes it a great tool for the global assessment of disease activity, the specific inflammatory phenotypes in psoriatic arthritis, and avert anatomical damage and physical functional disability.

When there is a suspicion of loss of efficacy from a biological agent, and hesitancy to change the biologic (due to cost and restrictions on access), ultrasonography could help add weight to decision making when considering changing a biologic agent. This was certainly evident in our case and helped the patient achieve more effective disease control.

**Key learning points/Conclusion:**

There is clear evidence that the introduction of biologic treatments offered the possibility of controlling multiple aspects of psoriatic arthritis using a single drug, minimising the need for additional therapies. Conventional treatments for psoriatic arthritis have limited efficacy for nail disease, enthesitis or axial involvement.

The sequential use of biologics has shown good effectiveness in successful treatment of moderate- severe cases and refractory psoriasis. Moreover, it has better control of signs/symptoms and offers improvement in the quality-of-life and overall functional outcome. Psoriatic patients have shown higher persistence rates with both first and second line drugs.

Although effective in a large proportion of patients, primary and secondary failures of anti-TNF-alpha agents have been well witnessed. Switching from one anti-TNF-alpha agent to a second (or even third) anti-TNF-alpha therapy has become a successful strategy of addressing treatment failures within this drug class.

Studies have proven that switching to a second TNF-alpha inhibitor is a strong therapeutic strategy. Interestingly, among the second line drugs, Secukinumab is found to be superior against adalimumab, leflunomide and ustekinumab.

Clinical assessment has limitations to assess psoriatic pathology, thus musculoskeletal ultrasound has become very useful in recent years to detect inflammation and inform clinical decisions. Unique US findings in psoriatic arthritis are paratenontis and enthesitis. Detection of specific features in psoriatic arthritis may help identify treatment failures and help better inform decisions about treatment change especially in the context of biologic sequential use.

